# Forward Genetic Screening Identifies a Small Molecule That Blocks *Toxoplasma gondii* Growth by Inhibiting Both Host- and Parasite-Encoded Kinases

**DOI:** 10.1371/journal.ppat.1004180

**Published:** 2014-06-12

**Authors:** Kevin M. Brown, Elena Suvorova, Andrew Farrell, Aaron McLain, Ashley Dittmar, Graham B. Wiley, Gabor Marth, Patrick M. Gaffney, Marc Jan Gubbels, Michael White, Ira J. Blader

**Affiliations:** 1 Department of Microbiology and Immunology, University of Oklahoma Health Sciences Center, Oklahoma City, Oklahoma, United States of America; 2 Departments of Molecular Medicine & Global Health, University of South Florida, Tampa, Florida, United States of America; 3 Department of Biology, Boston College, Chestnut Hill, Massachusetts, United States of America; 4 Department of Microbiology and Immunology, University at Buffalo, Buffalo, New York, United States of America; 5 Arthritis and Clinical Immunology Research Program, Oklahoma Medical Research Foundation, Oklahoma City, Oklahoma, United States of America; Stanford University, United States of America

## Abstract

The simultaneous targeting of host and pathogen processes represents an untapped approach for the treatment of intracellular infections. Hypoxia-inducible factor-1 (HIF-1) is a host cell transcription factor that is activated by and required for the growth of the intracellular protozoan parasite *Toxoplasma gondii* at physiological oxygen levels. Parasite activation of HIF-1 is blocked by inhibiting the family of closely related Activin-Like Kinase (ALK) host cell receptors ALK4, ALK5, and ALK7, which was determined in part by use of an ALK4,5,7 inhibitor named SB505124. Besides inhibiting HIF-1 activation, SB505124 also potently blocks parasite replication under normoxic conditions. To determine whether SB505124 inhibition of parasite growth was exclusively due to inhibition of ALK4,5,7 or because the drug inhibited a second kinase, SB505124-resistant parasites were isolated by chemical mutagenesis. Whole-genome sequencing of these mutants revealed mutations in the *Toxoplasma* MAP kinase, TgMAPK1. Allelic replacement of mutant TgMAPK1 alleles into wild-type parasites was sufficient to confer SB505124 resistance. SB505124 independently impacts TgMAPK1 and ALK4,5,7 signaling since drug resistant parasites could not activate HIF-1 in the presence of SB505124 or grow in HIF-1 deficient cells. In addition, TgMAPK1 kinase activity is inhibited by SB505124. Finally, mice treated with SB505124 had significantly lower tissue burdens following *Toxoplasma* infection. These data therefore identify SB505124 as a novel small molecule inhibitor that acts by inhibiting two distinct targets, host HIF-1 and TgMAPK1.

## Author Summary

Understanding how a compound blocks growth of an intracellular pathogen is important not only for developing these compounds into drugs that can be prescribed to patients, but also because these data will likely provide novel insight into the biology of these pathogens. Forward genetic screens are one established approach towards defining these mechanisms. But performing these screens with intracellular parasites has been limited not only because of technical limitations but also because the compounds may have off-target effects in either the host or parasite. Here, we report the first compound that kills a pathogen by simultaneously inhibiting distinct host- and parasite-encoded targets. Because developing drug resistance simultaneously to two targets is less likely, this work may highlight a new approach to antimicrobial drug discovery.

## Introduction

*Toxoplasma gondii* infects approximately one-third of the world's population and causes disease primarily in developing fetuses or immunocompromised individuals [1]. Humans and other intermediate hosts are infected with *Toxoplasma* by digesting either sporozoite-containing oocysts that are shed in feline fecal matter or bradyzoite-laden tissue cysts in undercooked meat [2]. In the intestine, the parasites infect intestinal epithelial cells and then convert into the replicative form of the parasite called tachyzoites [3]. As tachyzoites disseminate through the host, they encounter various host defenses or pharmacological agents that in most cases kill the tachyzoites. However, some escape killing and transform into bradyzoites that go on to develop into tissue cysts. These tissue cysts cannot be detected by the host's immune system and are impervious to most, if not all, currently prescribed drugs [3]–[8]. Thus, *Toxoplasma* is highly prevalent in humans, in large part, because tachyzoites have evolved to grow within its host until it is challenged and then respond by forming a life-long chronic infection.

For tachyzoites to be able to grow, they must establish a replicative niche within their host cells and do so by inducing a number of changes to host cell signaling, transcription, and organellar/cytoskeletal organization [9]–[17]. One of these changes includes activation of the host cell transcription factor hypoxia-inducible factor 1 (HIF-1), which is important for parasite growth [18]. *Toxoplasma* activates HIF-1 by stabilizing the abundance of the HIF-1α subunit. HIF-1α stabilization is accomplished by the parasite down regulating the abundance and activity of the Prolyl Hydroxylase Domain 2 (PHD2) protein whose primary function is to target HIF-1α for proteasomal-dependent degradation [19], [20]. To down regulate PHD2, *Toxoplasma* requires signaling via a family of host serine/threonine kinase receptors named the activin-like kinases (ALK4, ALK5, and ALK7) [21].

SB505124 [2-(5-benzo[1,3]dioxol-5-yl-2-tert-butyl-3H-imidazol-4-yl)-6-methylpyridine hydrochloride], which is a highly selective ALK4,5,7 competitive inhibitor, was one reagent used to demonstrate a role for ALK4,5,7 signaling in HIF-1 activation [21], [22]. But besides inhibiting ALK4,5,7/HIF-1, SB505124 also potently blocked parasite replication. Although supporting a role for ALK4,5,7 signaling in *Toxoplasma* growth, these data were enigmatic because the drug's effect on parasite growth was more severe than the loss of HIF-1α. For example, the drug significantly blocked parasite replication at 21% O_2_ (Figure 6 in [21]) whereas parasite growth was more modestly attenuated in HIF-101629226835445KO cells at this O_2_ tension (Figure 5 in [18]). Two plausible explanations exist to address this: first, ALK4,5,7 signaling regulates not only PHD2/HIF-1 but other host pathways that are important for parasite growth. Second, the drug has a second target that may be either host- or parasite-encoded. These possibilities were addressed using a forward genetic screen to isolate and characterize SB505124 resistant parasites. This screen revealed that SB505124 inhibits *Toxoplasma* growth by not only inhibiting ALK4,5,7/HIF-1 signaling but by targeting a parasite MAP kinase named TgMAPK1.

## Results

### Isolation of SB505124-Resistant Parasites

**Figure 1:**
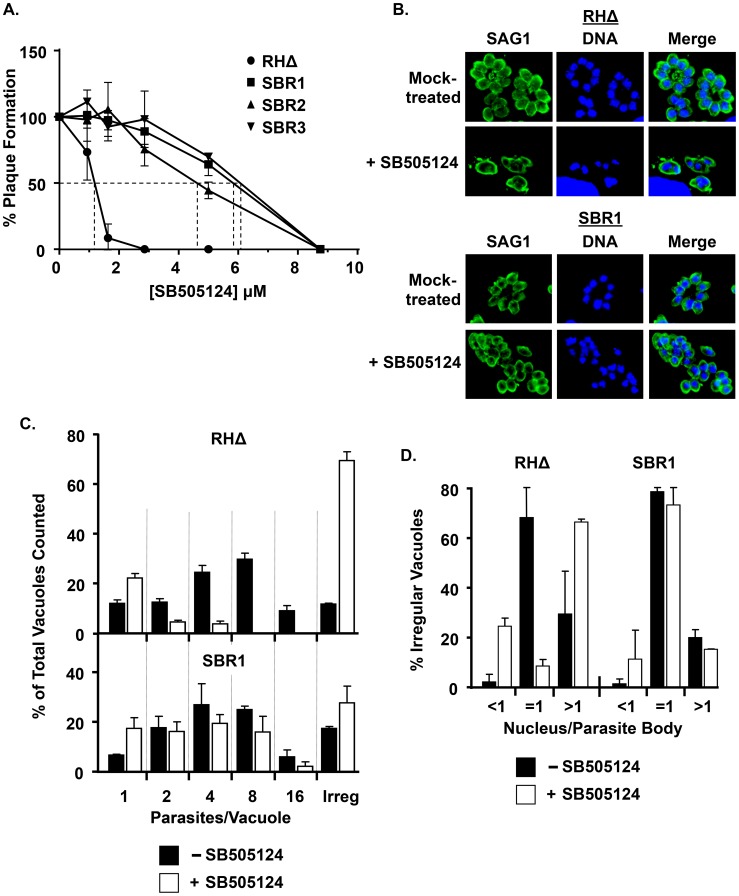
Generation of SBR mutants. A. Relative plaque formation in HFFs was determined for each parasite strain in the presence of increasing concentrations of SB505124. B–D. Parasite replication was measured by infecting HFFs on glass coverslips in the presence or absence of 3 µM SB505124 and then fixing the cells 24 hours later. Parasites and nuclei were detected with anti-SAG1 antibody and DAPI, respectively. B. Representative images. C. For each replicate, 100 vacuoles were monitored for parasites per vacuole and nuclei per parasite. Vacuoles were designated as being irregular if they contained an irregular number of parasites/vacuole (non 2^n^). Shown are averaged percentages and standard deviations of 2 independent experiments with two replicates each. D. Averaged percentages and standard deviations of irregular vacuoles (show in C) by nuclei per parasite.

To define how SB505124 inhibited parasite growth, we developed a forward genetic screen to isolate SB505124-resistant (SBR) parasites. Wild-type RHΔHXGPRT (RHΔ) parasites were chemically mutagenized with N-Ethyl-N-nitrosourea (ENU) and SBR mutants were isolated by growth in the presence of SB505124. Three SBR mutants (SBR1, SBR2, and SBR3) were isolated from three independent mutagenesis screens and relative resistance of each mutant to SB505124 was determined by plaquing assays. SBR mutants displayed similar IC_50_ values ranging from 4.9 µM to 5.4 µM, which relative to the parental RHΔ strain represented ∼5-fold increases in resistance to SB505124 (Figure 1A). Unless stated otherwise, the remaining assays in this report were performed with SBR1.

To define how SB505124 affected parasite growth, mock- or drug-treated parasites were stained with DAPI and anti-SAG1 antisera to assess DNA content and identify individual parasites by immunofluorescence. SAG1 staining revealed that, as we previously reported [21], 3 µM SB505124 caused parasites to arrest growth as single SAG1^+^ parasites (Figures 1B&C). However, DAPI staining indicated that most vacuoles, including the seemingly single SAG1^+^ parasites, contained irregular numbers (non-2^n^) of parasites/vacuole (Figure 1C “Irregular Vacuole”). Numbers of nuclei/parasite bodies of those parasites growing within the “Irregular Vacuoles” were quantified (Figure 1D). Approximately 65% of vacuoles from the drug-treated RHΔ parasites contained ≥2 nuclei/parasite body. We also noted that approximately 25% of the SB505124-treated vacuoles contained parasites that were SAG1^+^/DAPI^−^, which are denoted as <1 nucleus/parasite body. In contrast, SBR1 replication was neither affected by SB505124 nor did the drug trigger an accumulation of multinucleated parasites or irregular vacuoles (Figure 1C&D). These data suggest that SB505124 disrupts tachyzoite cell cycle progression.

SB505124 is a substituted imidazole compound that was developed from the same structural scaffold as the p38 MAP kinase inhibitor SB203580 [22]. SB203580 inhibits *Toxoplasma* replication and does so presumably by targeting a SB203580-sensitive, parasite-encoded kinase [23], [24]. Even though SB505124 more potently inhibits ALK4,5,7 than human p38 MAP kinases [22], it was possible that SB505124 reduced parasite growth by targeting the same kinase that SB203580 inhibits. A plaquing growth assay, however, revealed that SB203580 similarly inhibited RHΔ and SBR1 growth (IC_50_∼15 µM) (Supplemental Figure S1), which is consistent with earlier reports [23]. Thus, these two structurally related kinase inhibitors appear to inhibit *Toxoplasma* through distinct mechanisms and target proteins.

**Figure 2:**
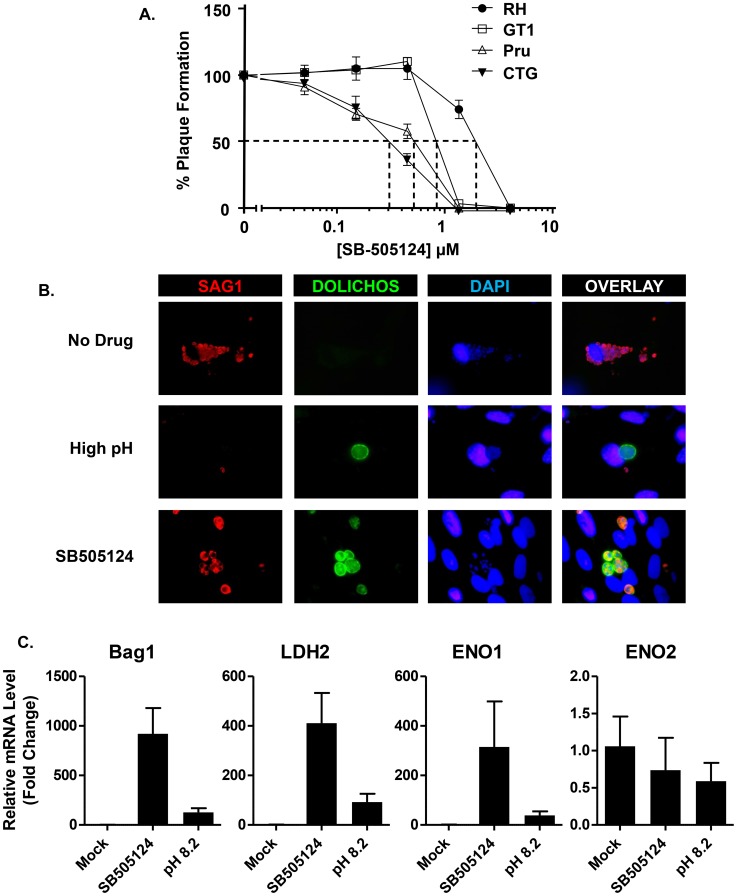
SB505124 induces bradyzoite development. A. HFFs growing in 24 well plates were infected with RH, GT1, Pru, and CTG. After 5–7 days, monolayers were methanol-fixed, stained with crystal violet, and plaques were counted. Means and standard deviations are shown. B. HFFs grown on glass coverslips were infected with Pru strain parasites and either mock-treated or treated with pH 8.2 media or 3 µM SB505124. After 72 h, cells were fixed and stained with anti-SAG1 antisera and Dolichos-FITC to visualize cysts. Shown are representative images. C. HFFs were infected with Pru and either mock-treated or treated with pH 8.2 media or 3 µM SB505124. After 72 h, parasites were released from host cells, washed, and lysed. Total RNA was extracted, DNase I-treated and converted to cDNA. Relative amounts of transcripts were determined by Real Time PCR using β-actin as an internal reference. Fold change for each gene's transcript abundance is reported as 2^−ΔΔCt^. Shown are averaged data with standard deviations from 3 experiments. Note that changes in ENO2 are not statistically significant.

The preceding assays were performed with RH strain parasites because it is the strain best suited for genetic manipulation. But one limitation of the RH strain is that it grows exclusively as tachyzoites and does not readily undergo bradyzoite differentiation either *in vitro* or *in vivo* as other parasite strains can. Before testing the effect of SB505124 on the ability of the other parasite strains to undergo bradyzoite development, we first assessed whether SB505124 inhibited their proliferation as tachyzoites. We therefore grew Pru (a Type II strain), CTG (a Type III strain), and GT-1 (a Type I strain that can form bradyzoites) tachyzoites in the presence of SB505124 and determined the drug's IC_50_ towards each strain (Figure 2A). SB505124 blocked lytic replication of all 3 strains with IC_50_ values lower than RH's IC_50_.

To assess the impact of SB505124 on bradyzoite development, GT-1, Pru, and CTG tachyzoites were grown on coverslips in the presence of SB505124. After 72 hours, parasites were fixed and stained with Dolichos-FITC to detect bradyzoite-containing cysts. We found that 3 µM SB505124 induced the formation of Dolichos^+^ cysts (Figure 2B and Supplemental Figure S2). But unlike high pH-induced bradyzoites, SAG1 staining could still be detected in the encysted parasites. To further assess bradyzoite development, Pru parasites were grown for 72 h in human foreskin fibroblasts (HFFs) in the presence of 3 µM SB505124 and bradyzoite specific gene expression was assessed by real time PCR. The data indicated that SB505124 induced a several hundred-fold increase of the bradyzoite gene transcripts, BAG1, LDH2, and ENO1 (Figure 2C). While a down-regulated trend was observed for the tachyzoite-specific gene ENO2, decreases in its abundance were not statistically significant (Figure 2C). Thus, SB505124 treatment induces some, but not all, features of bradyzoite development in cystogenic strains of *Toxoplasma*.

### TgMAPK1 Is a SBR Gene

**Figure 3:**
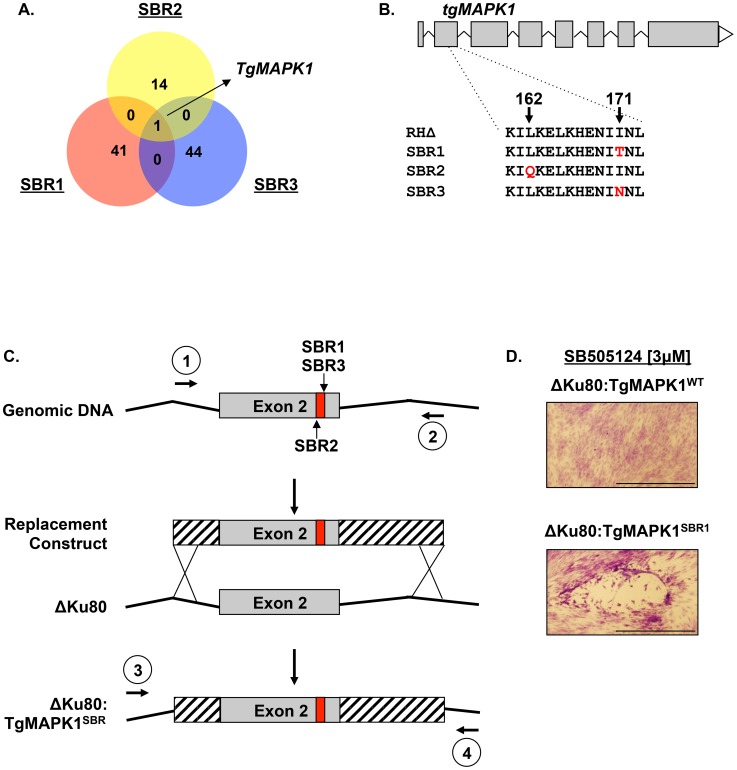
TgMAPK1 is an SBR gene. A. Venn diagram of whole genome sequencing data of codon-changing SNVs identified in each mutant. B. Amino acid positions of TgMAPK1^SBR^ mutations. C. TgMAPK1^SBR^ allelic replacement strategy. Primers 1 and 2 were used to amplify 944 bp fragments of genomic DNA containing the SBR allele and cloned into pCR2.1. Primers 3 and 4 were used to amplify 1055 bp fragments of genomic DNA to confirm allelic replacement by Sanger sequencing. D. RHΔku80 parasites were transfected with linearized TgMAPK1^WT^ or TgMAPK1^SBR^ replacement constructs and grown in 3 µM SB505124-treated HFFs. Shown are representative images depicting the ability of RHΔKu80:TgMAPK1^SBR1^ to grow and form plaques after 5 days of growth in the presence of 3 µM SB505124.

To identify the genetic basis for SB505124 resistance, genomic DNA purified from each SBR mutant and the parental RHΔ parasites were subjected to Illumina whole genome sequencing. The sequenced genomes were aligned to the GT-1 *Toxoplasma gondii* reference genome and compared to identify ENU-induced single nucleotide variants (insertions or deletions were not detected) (Supplementary Table S1). To prioritize testing of candidate SBR mutations, we searched for genes that were mutated in more than one SBR mutant. From this filter, only one gene, *Toxoplasma* MAP Kinase 1 (TgMAPK1; TGGT1_312570 @ www.toxodb.org), was mutated in all three SBR mutants (Figures 3A&B and Supplemental Table S1). These three mutations affected the translation of two amino acids (Leu162→Gln and Ile171→Thr or Asn) in exon 2 that are part of the kinase's predicted ATP binding pocket within its catalytic domain.

To determine whether these TgMAPK1 mutations were *bona fide* SBR alleles, we used an allelic replacement strategy to test whether replacing the wild-type TgMAPK1 allele with the mutant one conferred SB505124 resistance (Figure 3C). Thus, RHΔku80 parasites were transfected with TgMAPK1^WT^ or TgMAPK1^SBR1^ allelic replacement constructs consisting of 900 bp fragments of the TgMAPK1 gene containing either WT or candidate SBR alleles. The transfected parasites were grown in the presence of 3 µM SB505124 and parasite growth was monitored visually. While we could not detect growth of those parasites that were transfected with TgMAPK1^WT^ DNA, we found that parasites grew when they received any of the candidate SBR alleles (Figure 3D). To verify that the mutant TgMAPK1 alleles had properly integrated into the genome, we confirmed the presence of each SBR allele by sequencing a PCR fragment containing TgMAPK1 mutations (data not shown). Thus, single nucleotide mutations in exon 2 of TgMAPK1 were sufficient to rescue growth in SB505124.

### SB505124 Impacts TgMAPK1 and Host HIF-1 through Distinct Pathways

**Figure 4:**
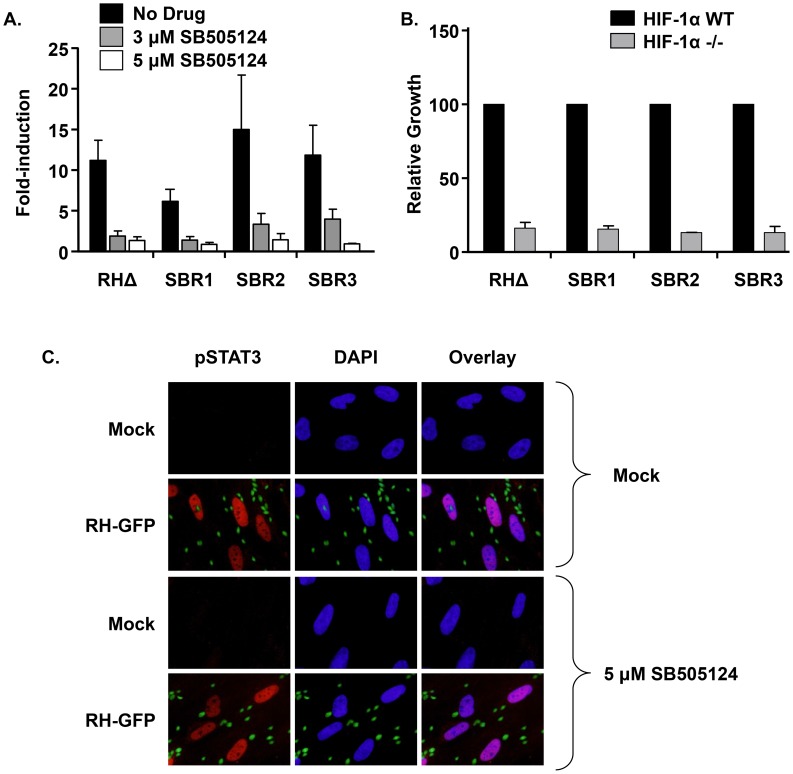
SB505124 impacts HIF1 and TgMAPK1 through distinct pathways. A. HIF-1 luciferase reporter activity in mock- or SB505124-treated MEFs was measured after 18 h of infection with RHΔ or SBR1-3. Shown are averaged measurements and standard deviations from at least 3 independent experiments performed in triplicate. B. HIF-1αWT and HIF-1α-/- MEFs were grown in 24 well plates and infected with RHΔ or SBR1-3. The plates were grown for 66 h at 3% O_2_ and then 5 µCi ^3^H-Uracil was added to each well to assess parasite growth. Shown are averaged data and standard deviations from 3 independent experiments performed in duplicate. C. HFFs grown on glass coverslips were infected with RH GFP at an MOI of 10 for 6 h in the presence or absence of 5 µM SB505124. Representative images are shown.

We hypothesized that if the function of TgMAPK1 was epistatic to ALK4,5,7/HIF-1 then HIF-1 activation would be unimpaired in the presence of SB505124 and that growth of the SBR mutants would be restored in HIF-1 knockout cells. First, HIF-1 luciferase reporter-transfected host cells were infected for 18 h with wild-type RHΔ, SBR1, SBR2, or SBR3 parasites in the absence or presence of SB505124. The data indicated that HIF-1 was activated by all 4 strains and this activation was similarly inhibited by SB505124 (Figure 4A). Next, we used [^3^H]-uracil incorporation to measure SBR mutant growth in HIF-1α knockout cells at 3% O_2_ and found that growth of the SBR mutants were as reduced as wild-type parasites growing in the HIF-1αKO cells (Figure 4B). HIF-1 is activated by a diffusible factor released from extracellular tachyzoites [18]. We, therefore, needed to eliminate the possibility that SB505124 blocked HIF-1 activation due to a global inhibition of host cell responses to extracellular parasites. Thus, we tested whether SB505124 inhibited STAT3 activation since activation of this host cell transcription factor is due to ROP16, which is a polymorphic rhoptry-encoded kinase that is injected by Type I and III strain parasites into host cells independently of invasion [15]. We found that RH strain-induced STAT3 phosphorylation and nuclear localization was unaffected by SB505124 (Figure 4C). Together, these data indicate that SB505124 has two distinct targets in *Toxoplasma*-infected cells – TgMAPK1 and host HIF-1.

### SB505124 Inhibits TgMAPK1 Autokinase Activity

**Figure 5:**
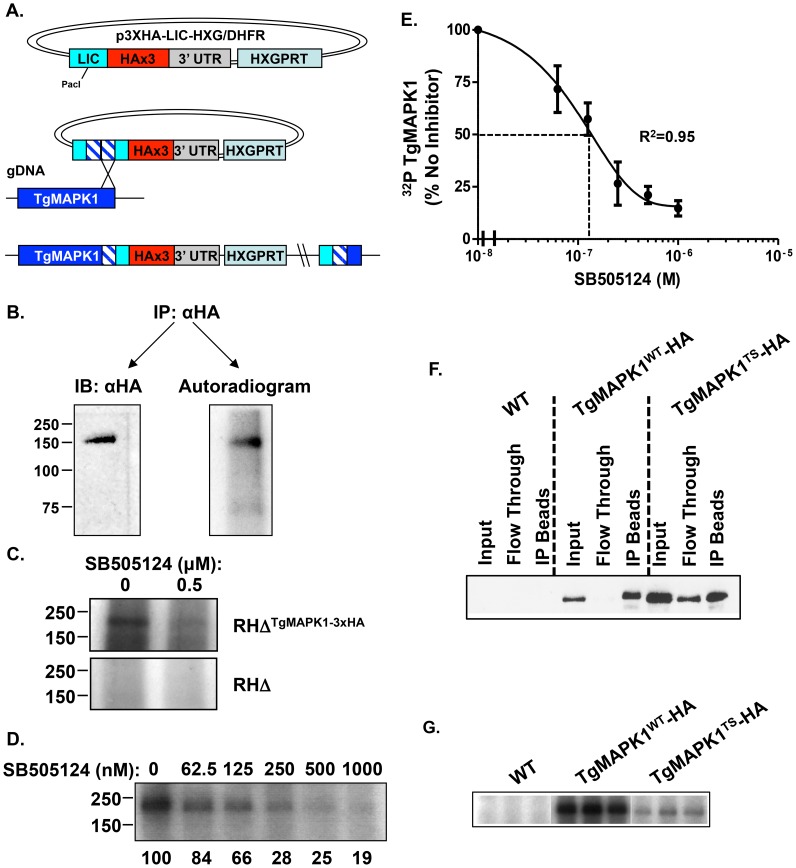
SB505124 directly targets TgMAPK1. A. Strategy for endogenously tagging TgMAPK1 with 3×HA tag. B. Immunoprecipitated TgMAPK1-HA was separated by SDS-PAGE for western blotting antibody and *in vitro* kinase assays. C. Equivalent volumes of RH WT or RH:TgMAPK1-HA lysate were added anti-HA sepharose beads, then washed and then processed for *in vitro* autokinase assays. The lysates were then separated by SDS-PAGE and visualized by autoradiography. Shown is a representative assay. D. Equivalent amounts of TgMAPK1-HA was immunoprecipitated from RH:TgMAPK1HA lysates using anti-HA sepharose beads and processed for *in vitro* kinase assays in the presence of increasing concentrations of SB505124. A representative assay with relative amounts of TgMAPK1 activity in each reaction is shown. E. Dose response curve showing averaged data and standard deviations from 3 experiments. F. Lysates (80 µg) were prepared from RHΔKu80ΔHPT (WT), RHΔKu80ΔHPT:TgMAPK1^WT-HA^, and RHΔKu80ΔHPT:TgMAPK1*^ts^* ^-HA^ parasites grown at 34°C. Epitope-tagged TgMAPK1 was then detected using rat anti-HA antisera in the whole cell lysate and the flow through and immunoprecipitates following immunoprecipitation using rabbit anti-HA antibody conjugated beads. G. Immunoprecipitates of the indicated HA-tagged TgMAPK1 alleles were washed in kinase assay buffer and then incubated with γ^32^P-ATP for 60′ at 34°C. Shown are triplicate samples prepared from the same lysates immunoprecipitated in F. The experiment was repeated 3 independent times and representative gels are shown.

SB505124 was identified as an ATP competitive inhibitor of ALK4,5,7 kinase activity [22]. Although SB505124 does not inhibit host MAPKs [22], we tested whether TgMAPK1 was identified as an SBR gene due to the drug inhibiting the parasite kinase. Our (and others [25]) initial attempts to express in *E. coli* either full-length TgMAPK1 or the kinase's N-terminal half containing the kinase domain resulted in a protein preparation of low purity and limited specific activity (not shown). Moreover, we were unable to eliminate the possibility that any activity we did detect was due to a bacterial kinase contaminating our preparations. We therefore developed a *Toxoplasma* strain in which the C-terminus of TgMAPK1 is epitope tagged at its endogenous chromosomal locus (Figure 5A). Western blotting whole cell lysates with anti-HA antibodies revealed a single immunoreactive band at ∼150 kDa, which is consistent with the predicted molecular weight of TgMAPK1-HAx3 of 141 kDa (Figure 5B). Unlike Brumlik et al [26], we failed to note the presence of smaller immunoreactive bands.

Because TgMAPK1 target substrates have yet to be identified, we assessed its activity using an autokinase assay. Thus, HA-tagged TgMAPK1 was immunoprecipitated from *Toxoplasma* lysates and *in vitro* kinase assays were performed in the presence of γ^32^P-ATP. We found that TgMAPK1 was autophosphorylated (Figure 5B), indicative of an active kinase, and that this autokinase activity was not detected in immunoprecipitates from wild type non-transgenic controls (Figure 5C). Dose response curves revealed that SB505124 inhibited TgMAPK1 autokinase activity with an apparent IC_50_ of 125 nM (Figure 5D&E).

To address the possibility that the TgMAPK1 immunoprecipitates contained a contaminating kinase whose activity was SB505124 sensitive, we attempted to ectopically express a kinase-dead TgMAPK1 mutant cDNA by mutating the conserved lysine^140^ in the catalytic domain to an arginine. Repeated attempts to either transiently or stably express this mutant were unsuccessful as were attempts to express SBR mutants that were epitope tagged as either transgenes or at the endogenous TgMAPK1 locus. We therefore turned our attention to a TgMAPK1 temperature sensitive mutant that was isolated independently of this study in a temperature sensitive growth screen (LE and MW; manuscript in preparation). To minimize potential misfolding of the TgMAPK1^ts^ protein, autokinase activity was compared between parasites grown at 34°C (the permissive growth temperature for the *ts* mutant) that harbored either HA-tagged TgMAPK1^WT^ or TgMAPK1^ts^ alleles. Unlike TgMAPK1 kinase-dead and SBR mutants, TgMAPK1^ts-HA^ could be stably expressed and similar amounts of TgMAPK1^WT-HA^ or TgMAPK1^ts-HA^ could be immunoprecipitated using anti-HA beads (Figure 5F). In contrast, significantly lower levels of autokinase activity could be detected in the TgMAPK1^ts^ immunoprecipitates suggesting that the kinase activity in the immunoprecipitates was most likely dependent on TgMAPK1 and not a contaminating kinase (Figure 5G). Even though the wild-type and ts mutants grew at similar rates at 34°C we cannot fully rule out the possibility that potential misfolding of TgMAPK1^ts^ at the permissive temperature may affecting binding of a contaminating kinase. The differences in autokinase activity between the wild-type and *ts* kinases and the fact that the SBR mutations are in the region of the ATP binding pocket where the SB class of kinase inhibitors interact with their target kinases [27]–[29] strongly suggests that TgMAPK1 is an active kinase that can be inhibited by SB505124.

### SB505124 Reduces Parasite Tissue Burden

**Figure 6:**
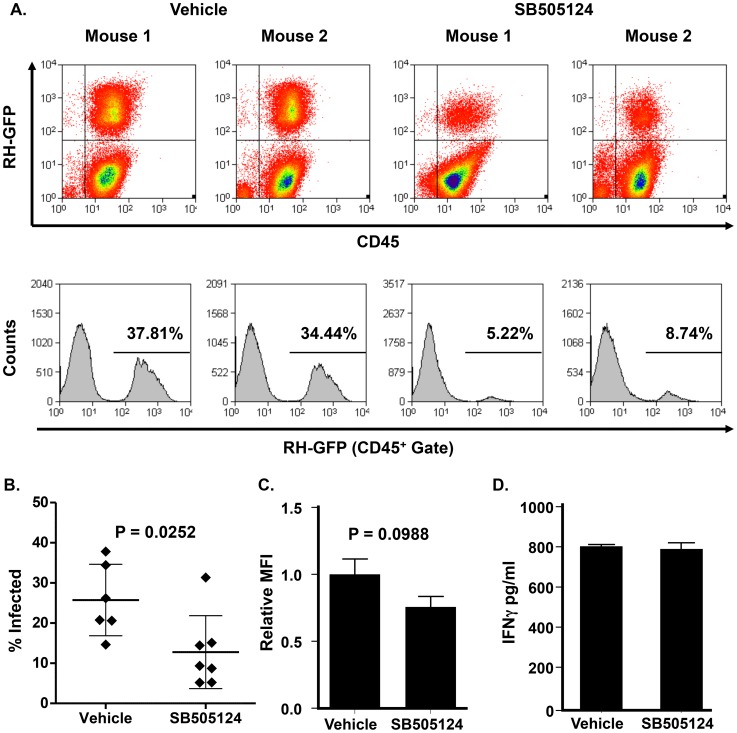
SB505124 reduces parasite growth in *Toxoplasma*-infected mice. RH-GFP infected mice were intraperitoneally injected daily with 10 mg/kg SB505124 or DMSO alone. After 5 days post-infection, mice were sacrificed and flow cytometric analysis was performed on peritoneal exudate cells (3–4 mice per treatment group per experiment, 2 independent experiments). A. FACS plots (upper) and histograms (lower) showing percentages of infected (GFP^+^) cells of two representative mice per treatment group. B. Mean percentages of infected cells between treatment groups with standard deviations. C. Relative MFI of infected (GFP^+^) cells with standard deviations. D. ELISA determination of serum IFNγ levels of mock- and drug-treated, intraperitoneally infected mice 5 days post-infection. Shown are average and standard deviations.

Our *in vitro* data suggests that SB505124 blocks *Toxoplasma* growth in an unusual manner by which both host and parasite pathways are simultaneously targeted. To assess whether SB505124 can limit parasite growth *in vivo*, C57Bl/6 mice were intraperitoneally infected with 10^3^ GFP-expressing RH tachyzoites and then treated with daily intraperitoneal injections of either 10 mg/kg SB505124 or vehicle (DMSO). On Day 5 post-infection, mice were sacrificed and peritoneal exudate cells were stained with anti-CD45 (to identify infiltrating leukocytes) and analyzed by flow cytometry. Mice treated with SB505124 had significantly fewer numbers of infected cells (12.8%) compared to the vehicle controls (25.7%) as measured as GFP^+^/CD45^+^ events (p = 0.0252) (Figures 6A,B). To determine whether the infected cells contained similar numbers of parasites, we measured the mean fluorescence intensity (MFI) of GFP in CD45^+^/GFP^+^ cells and found that infected cells from SB505124-treated mice displayed a 26.8% reduction in relative GFP MFI compared to vehicle-treated control mice (p = 0.0988) (Figure 6C). Though this decrease was not statistically significant, it is likely an underestimation as SB505124-treated parasites display enlarged, translationally active parasite bodies that could still produce high levels of GFP. The polyploidy phenotype induced by SB505124 precluded us from further assessing parasite burdens by qPCR analysis of genomic DNA.

Since SB505124 inhibits TGFβ signaling, a known antagonist of IFNγ production [22], [30], it is possible that the effect of SB505124 on *Toxoplasma* in mice is due to enhanced IFNγ-driven immunity. However, IFNγ serum levels from the mice 5 days post infection were identical between SB505124 and vehicle-control treated mice (Figure 6D).

## Discussion

Our initial interest in SB505124 stemmed from finding that this compound blocked the parasite's ability to activate HIF-1 via ALK4,5,7 signaling [21]. This conclusion was supported by additional data showing that overexpression of SMAD7, which is an endogenous ALK4,5,7 inhibitor, also blocked parasite activation of HIF-1 [21]. Additional unpublished work demonstrated that HIF-1 activity is increased in cells transfected with ALK4,5,7 expression plasmids. But since parasite replication was more severely reduced in SB505124-treated cells than in HIF-1α knockout cells, these data suggested that in addition to the ALK4,5,7/HIF-1 pathway the drug had at least one additional target that could be in either the host or parasite. Our forward genetic screen resolved this issue and identified SB505124 as the first compound that we are aware of that inhibits growth of an intracellular pathogen by acting on seemingly unrelated targets in both the host and parasite. We believe that collectively our data demonstrate that TgMAPK1 and ALK4,5,7/HIF-1 are the two relevant SB505124 targets in parasite-infected cells. While we cannot rule out that the drug may have additional targets in *Toxoplasma*-infected cells, these targets would not be important for parasite growth.

Although the catalytic domain of TgMAPK1 is homologous to other MAPKs, it has two unusual features that may facilitate TgMAPK1-specific agonist development. First, TgMAPK1 has 3 unique insertions within its catalytic domain with the most predominant one being a 93 amino acid insertion between the DFGLAR-motif that coordinates divalent cations. How the catalytic domain of the kinase can properly fold with this large insertion is currently unknown and cannot be easily predicted since the insertion prevents protein-modeling programs from using known MAPK structures as scaffolds (Brown and Blader, unpublished results). Second, TgMAPK1 has an ∼800 amino acid C-terminal extension that lacks conserved domains and has no homology to any known protein either in *Toxoplasma* or its host. Given that inhibition of TgMAPK1 induces multinucleated parasites (suggesting a cell cycle defect) and that the C-terminal extension facilitates its localization to mitotic structures (E. Suvorovo et al, manuscript in preparation) we hypothesize that TgMAPK1 functions in cell cycle regulation and our future work will define if and how TgMAPK1 regulates the cell cycle.

TgMAPK1 was originally discovered based on its homology to human MAPKs [24]. It was assigned then as a p38 MAPK homolog, in part, because its catalytic activity was reported to be sensitive to SB203580. In our hands, TgMAPK1 activity was largely unaffected by this compound (Supplemental Figure S1B), although a general decrease in autokinase activity by low SB203580 levels was noted that may reflect reduced non-specific binding of ATP to the kinase. Regardless, higher amounts of SB203580 did not significantly decrease TgMAPK1 autokinase activity and SBR mutants are similarly sensitive to SB203580 (Supplemental Figure S1A&B). We believe that there are two primary reasons for differences in between our data and those of [24]. First, our experiments used endogenously epitope-tagged TgMAPK1 that was immunoprecipitated from tachyzoites. In contrast, Brumlik and colleagues used a bacterially expressed construct that only contained TgMAPK1's catalytic domain and most of this protein was expressed in bacterial inclusion bodies (our unpublished observations) [24]. Even though we could purify the protein from the inclusion bodies under denaturing conditions, very weak kinase activity was detected after refolding and we could not verify whether this protein was properly folded. Attempts to purify the soluble protein resulted in a preparation of poor purity (as noted by [24]) whose activity appeared to be due to a contaminating bacterial kinase since recombinant expression of a catalytically inactive kinase mutant had the same rate of activity as the wild-type kinase (not shown). Recently, Sugi et al demonstrated that the SBR2 (Leu162→Gln) and SBR3 (Ile171→Asn) alleles conferred resistance to the effect of the bumped kinase inhibitor 1NM-PP1 on parasite replication [25]. TgMAPK1 resistance to 1NM-PP1 is independent of its well-described inhibition of *Toxoplasma* Calcium-Dependent Protein Kinase 1, whose activity is required for parasite invasion and egress [31]. xxx But unlike our work, Sugi et al. did not test whether 1NM-PP1 affects parasite replication by directly inhibiting TgMAPK1 or if resistance emerged because TgMAPK1 functions downstream of the protein(s) that 1NM-PP1 interacts with [25], [32]. It is also noteworthy that we were unable to successfully express a kinase-dead mutant of TgMAPK1 as a transgene either in transiently or stably transfected parasites. The most likely explanation for this is that expression of the kinase dead TgMAPK1 mutant has a dominant negative effect either by binding its upstream activating kinase or its downstream targets.

The continuing emergence of antimicrobial resistance requires novel approaches to the design of new drugs and treatments. Although simultaneous inhibition of both host and parasite targets is an untested approach, one benefit would be that resistance to a compound that impacts a host cell target is less likely to develop [33]. As a proof of concept, we showed that SB505124 reduced tachyzoite burdens 5 days after mice were intraperitoneally infected. We did not examine later time points for three reasons. First, we demonstrated that SB505124 will induce bradyzoite development *in vitro* and therefore it is possible that long-term treatments with this drug would lead to increased cyst burden. Second, germline loss of functions mutations in HIF-1α causes an embryonic lethal phenotype in mice [34]. Thus, HIF-1α can only be deleted in specific cell types using cre-recombinase expressing mice. But, *Toxoplasma* infects and forms bradyzoites in multiple types of cells. Since tissues consist of heterogeneous populations of cells it would, therefore, be difficult to assess how a cell-specific loss of HIF-1α would impact bradyzoite development. Third, ALK5 is the TGFβ receptor and long-term inhibition of this key immunosuppressive cytokine could have a deleterious effect on mice independent of its role in regulating parasite growth.

Parasites treated with SB505124 developed into bradyzoites under *in vitro* conditions. Given that the drug induces RH parasites to become multi-nucleated (suggesting a role for TgMAPK1 in cell cycle), these data are consistent with earlier observations that bradyzoite development represents an exit from the cell cycle during the transition between S to M phase (*Toxoplasma* lacks a G2 phase) [35], [36]. One implication of bradyzoite development being potentially triggered by TgMAPK1 inhibition is that it may open the door to two novel but not necessarily mutually exclusive approaches for treating toxoplasmosis. First, we hypothesize that compounds that either act as TgMAPK1 agonists or mimic the activities of TgMAPK1 substrates would be predicted to act as inhibitors of bradyzoite development. Because bradyzoites are impervious to both immune surveillance mechanisms and anti-parasitic compounds [3]–[8], these small molecules would maintain the parasite as tachyzoites, which is a growth state that would prolong their susceptibility to currently prescribed drugs. Second we will focus on identifying a compound(s) that specifically blocks parasites from activating ALK4,5,7 or prevent these receptors from triggering HIF-1 activity. We believe that either of these approaches would be valid even if SB505124 induces bradyzoite development as a consequence of the drug activating a more generalized stress response. Our future analysis of TgMAPK1 regulation and substrate identification will resolve how TgMAPK1 influences bradyzoite differentiation.

In summary, we used a forward genetic screen to demonstrate that a serine/threonine kinase inhibitor blocks *Toxoplasma* growth through two distinct targets and our future work will focus on this issue. Kinase inhibitors are particularly useful for dual-target screens because even though they are designed to inhibit human kinases their off target effects cannot be predicted [37]. For example, we showed that SB203580 does not inhibit TgMAPK1, which bears homology to p38 MAPKs. Rather TgMAPK1 is inhibited by SB505124 even though this compound does not appear to significantly affect p38 MAPK activity [22]. Our work, therefore, also highlights the utility of repurposing drugs and investigative compounds originally developed to target cancer and other diseases for the study and/or treatment of microbial infections [38]–[41].

## Materials and Methods

### Ethics

Animal protocols (IACUC Protocol #11-075I) were approved by the University of Oklahoma Health Sciences Center IACUC. This study was carried out in strict accordance with the Public Health Service Policy on Humane Care and Use of Laboratory Animals and AAALAC accreditation guidelines.

### Cell and Parasites

*Toxoplasma* RH, RHΔHXGPRT, RHΔ-GFP (kindly provided by Dr. Gustavo Arrizabalaga of Indiana University), RHΔ RHΔHXGPRTΔKu80 (kindly provided by Dr. David Bzik of Dartmouth University), GT-1, Pru, and CTG parasites were maintained in human foreskin fibroblasts in Dulbecco's Modification of Eagle's Medium (DMEM) (Mediatech; Manassas, VA) supplemented with 10% fetal bovine serum (Mediatech), 2 mM L-glutamine (Mediatech), and 100 IU/ml penicillin – 100 µg/ml streptomycin (Mediatech). The TgMAPK1^ts-HA^ temperature sensitive (*ts*) TgMAPK1 mutant will be described in detail elsewhere (manuscript in preparation). Other host cell lines were maintained in the HFF growth medium. All host cell lines and parasites were routinely tested for *Mycoplasma* contamination with the MycoAlert Mycoplasma Detection Kit (Lonza, Basel, Switzerland) and found to be negative.

### Chemical Mutagenesis

Intracellular RHΔHXGPRT tachyzoites grown in T-75 flasks were mutagenized with 300 µg/ml ENU (Sigma; St. Louis, MO) in complete DMEM for 2 hours at 37°C as previously described [42], [43]. After ENU treatment, media was removed and the monolayer was washed four times with 1× phosphate-buffered saline. Mutagenized parasites were then released from host cells by scraping and syringe-lysis (27 g needle) and washed with 30 ml DMEM. Parasites were allowed to recover in fresh HFFs for 72 h and then grown in the presence of SB505124 (TGF-βRI Kinase Inhibitor III (EMD Millipore; Billerica, MA)). After several rounds of selection in SB505124, individual clones were obtained by limiting dilution in 96 well plates of HFFs. SBR1 was isolated from a population after 4 passages in 3 µM SB505124 and 11 passages in 5 µM SB505124. SBR2 was isolated after 4 passages in 5 µM SB505124. SBR3 was isolated after 5 passages in 5 µM SB505124.

### Growth Assays

Plaque assays were performed by adding 200 parasites to each well of a 24 well plate containing confluent HFFs. After 5–7 days, the monolayers were methanol-fixed and stained with 0.1% crystal violet. Plaques were counted using 4× magnification on an inverted dissecting microscope. Uracil incorporation assays were performed by growing parasites in 24 well plates. After 66 h, 5 µCi ^3^H-uracil (MP Biomedical; Santa Ana, CA) was added to the wells for an additional 6 h of the assay. Wells were washed, precipitated with 10% trichloroacetic acid, and ^3^H-uracil counted by liquid scintillation.

### Whole Genome Sequencing

Parasites were grown in confluent HFFs until the host monolayer had completely lysed. The media containing the extracellular parasites were collected without further scraping, washed with serum-free DMEM, passed three times through a 27 g needle, and then filtered through a 3 µm pore size filter membrane. Genomic DNA was isolated using the Qiamp DNA mini kit (Qiagen; Valencia, CA) using the manufacturer's protocol for cultured cells. qRT-PCR (not shown) determined that host DNA contamination was less than 0.5% and this result was confirmed by the whole genome sequencing data. Sequencing libraries were prepared from genomic DNA (1 µg) using the Truseq DNA LT Sample Prep Kit v2 as per the manufacturer's protocols (Illumina, San Diego, CA) and the libraries sequenced on an Illumina HiSeq 2000 instrument with 100 bp, paired-end reads. Between 30–37-fold coverage was achieved for all genomes. Genome sequences were aligned with the MOSAIK program [44], [45] using the type I parasite genome of GT-1 as a reference and single nucleotide variants identified with variant caller program FreeBayes [46]. For MOSAIK, the default parameters we used had a hash size of 14. For FreeBayes, we used the program's default parameters with the exception that: ploidy (-p) was set to 1, a P value cutoff of 0.9 (-P) was used, complex events were ignored (-u), and population priors were turned off (-no-population-priors) as they are not applicable to the *Toxoplasma* genome.

### Allelic Replacement of TgMAPK1^SBR^ Mutations

A 944 bp fragment of TgMAPK1 was PCR amplified using Platinum Pfx polymerase (Invitrogen; Carlsbad, CA) from wild-type and SBR mutant parasites with forward (5′-TGCATGGCGATGAGTTTCTGAACG-3′) and reverse (5′- TCGTGTCGACGTTTCTTCTGTGGA-3′) primers. PCR reactions were incubated with Taq polymerase (Invitrogen) for 10′ to add 3′ A-overhangs. PCR products were gel purified and cloned into pCR2.1 (Invitrogen) by TA ligation. Inserts were sequenced verified by conventional sequencing.

Parasites were transfected with 50 µg of linearized plasmid resuspended in cytomix buffer (2 mM ATP, 5 mM glutathione). For each transfection, 2×10^7^ RHΔHXGPRTΔKu80 tachyzoites were washed in cytomix buffer and resuspended in 0.5 ml complete cytomix buffer. The plasmid DNA was added to the parasites in a BTX electroporation cuvette (0.4 mm gap), and electroporated into the parasites at 2000 V, 50 ohm, 25 µF with a BTX ECM 360 (Holliston, MA). Parasites were then transferred to 75 cm^2^ flasks containing confluent HFF monolayers and after 72 h 3 µM SB505124 was added. Parasites receiving pCR2.1:TgMAPK1^WT^ failed to grow whereas those that received pCR2.1:TgMAPK1^SBR1-3^ were able to be passaged indefinitely in 3 µM SB505124. Individual clones were isolated by limiting dilution as described above and single clones were named RH:MAPK1^SBR1^, RH:MAPK1^SBR2^, and RH:MAPK1^SBR3^. To ensure that the SBR mutation was incorporated into the endogenous MAPK1 locus, PCR fragments were generated from genomic DNA using primers that flanked the region of the amplicon. The amplicon was then gel purified and cloned into PCR2.1. At least 5 independent transformants were sequenced via conventional Sanger sequencing using M13 forward and reverse universal primers.

### Fluorescence Microscopy

RHΔ or SBR1 tachyzoites were added to 24 well plates containing confluent HFFs on glass coverslips in the presence or absence of 3 µM SB505124. After 24 h, coverslips were methanol-fixed, blocked with 3% bovine serum albumin, and labeled with rabbit anti-SAG1 (1∶100000; kindly provided by Dr. John Boothroyd, Stanford University) followed by Alexa Fluor 488-conjugated goat anti-rabbit (Invitrogen). Coverslips were mounted to slides with Vectashield containing DAPI (Vector Lab; Burlingame, CA). One hundred randomly chosen vacuoles/coverslip were counted.

Phospho-STAT3 was detected in HFFs infected with RH-GFP (MOI of 10) tachyzoites for 6 h in the absence or presence of 5 µM SB505124. Cells were fixed with 4% formaldehyde, permeabilized with ice-cold methanol for 5 minutes, blocked at room temperature for 2 h with 3% BSA, and incubated with rabbit anti-phosphoSTAT3 (Cell Signaling; Danvers, MA) overnight at 4°C. After washing, cells were stained with Alexa Fluor 594 conjugated goat anti-rabbit (Invitrogen) and mounted to slides with Vectashield containing DAPI.

Bradyzoite cysts were detected in HFFs 72 h after adding either 3 µM SB505124 or pH 8.1 media [47]. Monolayers were fixed with ice-cold methanol for 5′ and then stained to detect SAG1 as described above. Bradyzoite cyst wall was detected by staining with 5 µg/ml FITC-conjugated Dolichos (Vector Labs). The frequency of Dolichos^+^ vacuoles was quantified by examining a minimum of one thousand vacuoles per strain from three independent experiments using a Cytation 3 (Biotek Instruments, Inc., Winooski, VT) cell imaging multi-mode reader at 20× magnification.

### *In Vitro* Kinase Assays

To generate strains in which TgMAPK1 is epitope tagged at its C-terminus with three HA repeats (RHΔHXGPRTΔKu80:TgMAPK1-3XHA), a 1513 bp fragment was amplified from the 3′ end of TgMAPK1 using primers TgMAPK1-3XHAF and TgMAPK1-3XHAR and cloned into p3xHA-LIC-HXGPRT by ligation independent cloning to create pTgMAPK1-3XHA [48]. RHΔHXGPRTΔKu80 parasites were transfected with PacI-linearized TgMAPK1 plasmids and transfectants selected with mycophenolic acid/xanthine. Proper integration and expression was by PCR (not shown), Western blotting (Figure 5B), and immunofluorescence (E. Suvorova et al., Manuscript in Preparation).

RHΔHXGPRTΔKu80:TgMAPK1^WT-HA^, RHΔHXGPRTΔKu80:TgMAPK1*^ts^* ^-HA^, or wild-type RHΔHXGPRTΔKu80 were grown overnight in confluent 75 cm^2^ flasks. Extracellular parasites were washed away and intracellular parasites were released by scraping and syringe lysis. The parasites were washed and lysed on ice with 200 µl modified RIPA buffer (50 mM Tris pH 7.4, 1% NP-40; 0.1% SDS, 500 mM NaCl) supplemented with 1× EDTA-free Protease Inhibitor Cocktail (Roche; Indianapolis, IN) per 10^7^ parasites. Lysates were centrifuged at 20,000×*g* for 15′ at 4°C to remove insoluble material and the supernatant was precleared with rabbit IgG-conjugated sepharose beads (Cell Signaling). Precleared lysates were added to 10 µl anti-HA-sepharose beads (Cell Signaling) and incubated overnight at 4°C, washed 3 times in modified RIPA buffer, and 1 time in kinase assay buffer (20 mM HEPES pH 7.48, 25 mM glycerophosphate, 25 mM MgCl_2_, 0.5 mM DTT, and 0.1 mM Na_3_VO_4_). Immune complexes were then evenly distributed between sample tubes (the equivalent of 200 µg of precleared lysate was used for each sample) and incubated with 10 µCi γ^32^P-ATP (MP Biomedicals) in kinase assay buffer at 34°C for 1 hour. Kinase reactions were stopped by adding 5 µl 6× sample buffer and boiling the samples for 5′. Reactions were separated by SDS-PAGE (10% acrylamide) and gels were fixed for 20′ in acetic acid∶methanol∶H_2_0 (1∶5∶4) and dried. The gels were exposed to film and analyzed using ImageJ software.

We attempted to express kinase-dead TgMAPK1 (K140R) and SBR mutants as a transgenes by synthesizing full length wild-type and mutant constructs in frame with C-terminal 3× HA-tags (Genescript; Piscataway, NJ). The cDNA constructs were cloned into pSAGCAT by replacing the CAT gene with the TgMAPK1 gene [49]. The constructs were then transfected into RH strain parasites and 24 h later the parasites were harvested and transgenic TgMAPK1 expression assessed by Western blotting and activity by *in vitro* kinase reaction. We were only able to detect low levels of mutant TgMAPK1 protein even when 175 cm^2^ flasks were used for the transfection and this amount of kinase was not sufficient for the autokinase reactions (not shown). In contrast, 25 cm^2^ flasks were sufficient to detect and assay the wild-type kinase. We next cloned the TgMAPK1 alleles into ptubYFPYFP/sagCAT [48] by replacing the YFP genes with the kinase alleles. Using chloramphenicol selection, stable transformants could be isolated for those parasites receiving wild-type TgMAPK1 but we could not obtain transformants for parasites receiving mutant alleles.

We also attempted to epitope tag the SBR mutants at their endogenous locus by cloning the 1.2 kb 3′ most region (up to the stop codon) of the genomic region of the TgMAPK1 into pSF-TAP-LIC-HXG in frame with the SF-TAP tag [48]. This single construct is compatible with both the wild type and SBR strains since the 3′ region of homology is downstream of the SBR mutations. We linearized the vector with NcoI, a unique restriction site within the TgMAPK1 sequence giving 677 bp of homology to the endogenous TgMAPK1 allele, and transfected the DNA into RHΔKu80ΔHXGPRT:*TgMAPK1^SBR1^* and the parental wild type strain. But, we were unable to recover viable SBR parasites from selection with MPA/xanthine even though we were able to do so for the wild-type parasites.

### Western Blotting

Proteins were separated by SDS-PAGE and transferred to PVDF membranes. Membranes were blocked with 5% BSA-TBST. Rat anti-HA (Roche) was used at a concentration of 1∶500. Goat anti-rat HRP (Cell Signaling) was used at a concentration of 1∶2000 and detected using Supersignal West Pico ECL reagent (Thermo, Waltham, MA) with a gel doc system.

### Reverse Transcriptase Real Time-PCR

RNA was isolated with the RNeasy Midi kit (Qiagen) using the manufacturer's instructions for cultured cells. RNA quality and yield was assessed by UV spectrophotometry and horizontal electrophoresis. Total RNA was treated with DNAse I (Invitrogen) and converted to cDNA using Superscript III (Invitrogen) using random hexamer primers. Relative abundances of bradyzoite gene transcripts BAG1, LDH2, ENO1 and tachyzoite transcript ENO2 were calculated with the 2^−ΔΔCt^ method [50] using *Toxoplasma* β-actin as an internal control (see Supplemental Table S2 for primer sequences).

### HIF1 Reporter Assay

HIF-1 luciferase assays were performed as previously described [18]. Briefly, murine embryonic fibroblasts were transfected pHRE-luc and pTK-Rel (as a transfection efficiency control) and 24 h later 2×10^6^ parasites were added to the wells in the presence or absence of SB505124. 18 hours later, luciferase activity was measured using the Dual-Glo luciferase assay kit (Promega, Madison, WI).

### SB505124 *In Vivo* Experiments

C57Bl/6 female mice were infected intraperitoneally with 10^3^ freshly purified RH-GFP parasites in a volume of 200 µl phenol-red-free DMEM. Beginning 1 h post infection, mice received 10 mg/kg SB505124 or DMSO alone intraperitoneally once daily. The drug had no apparent effect on the health of the animals during this time course. On day 5 post-infection, mice were sacrificed by CO_2_ asphyxiation and PECs collected in a 5 ml PBS-lavage. Cells were washed, stained with anti-mouse APC-conjugated CD45, and analyzed by flow cytometry using a FACSCalibur cytometer (BD Biosciences, San Jose, CA). 10^5^ events were counted and subsequent analysis was performed using Summit (Dako, Carpinteria, CA). IFNγ was measured using Mouse IFNγ ‘Femto-HS’ High Sensitivity ELISA (eBioscience; San Diego, CA).

## Supporting Information

Figure S1 A. RHΔ or SBR1 parasites were grown in HFFs with the indicated concentrations of SB203580. After 5 days, the cells were fixed and numbers of plaques formed were determined by crystal violet staining. Shown are averages and standard deviations of 3 independent experiments performed in triplicate. B. Equivalent amounts of TgMAPK1-HA was immunoprecipitated from RH:TgMAPK1HA lysates using anti-HA sepharose beads and processed for *in vitro* kinase assays in the presence of increasing concentrations of SB203580.
(TIF)

Figure S2 HFFs grown on glass coverslips were infected with GT-1 (A) or CTG (B) strain tachyzoites and then either mock-treated or treated with pH 8.2 media or 3 µM SB505124. After 72 h, cells were fixed and stained with anti-SAG1 and Dolichos-FITC to visualize cysts. Shown are representative images. C. Quantification of numbers of GT-1, Pru, and CTG strain parasites thath were Dolichos (DBA)^+^ after exposure to SB505124. Note that images for Pru strain parasites are shown in Figure 2B.
(TIFF)

Table S1 Summary of the whole genome sequencing of the parental and SBR mutants. The first sheet is a summary of the sequencing for each strain and the number of base pairs sequenced for parasite nuclear and mitochondrial genomes as well as the host genome. The other sheets list the position of each identified mutation, how the mutation affected the a change in protein sequence, and, if applicable, the gene in which the mutation lies.
(XLS)

Table S2 List of either PCR primers to clone TgMAPK1 constructs or of RT-PCR primers to assess bradyzoite gene expression.
(DOCX)
