# Supplementary figures and images for: Forward Genetic Screening Identifies a Small Molecule That Blocks Toxoplasma gondii Growth by Inhibiting Both Host- and Parasite-Encoded Kinases
Source: PLoS Pathog. 2014 Jun 12;10(6):e1004180. doi: 10.1371/journal.ppat.1004180 (PMC4055737; doi:10.1371/journal.ppat.1004180)

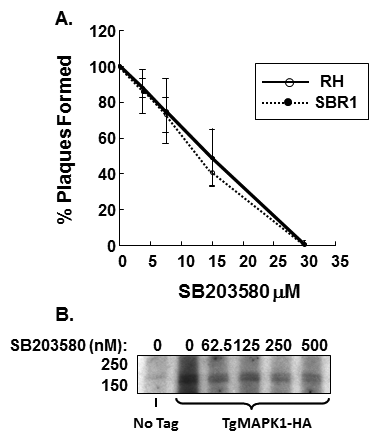

Supplement: Figure S1 — A. RHΔ or SBR1 parasites were grown in HFFs with the indicated concentrations of SB203580. After 5 days, the cells were fixed and numbers of plaques formed were determined by crystal violet staining. Shown are averages and standard deviations of 3 independent experiments performed in triplicate. B. Equivalent amounts of TgMAPK1-HA was immunoprecipitated from RH:TgMAPK1HA lysates using anti-HA sepharose beads and processed for in vitro kinase assays in the presence of increasing concentrations of SB203580. (TIF) [file ppat.1004180.s001.tif]

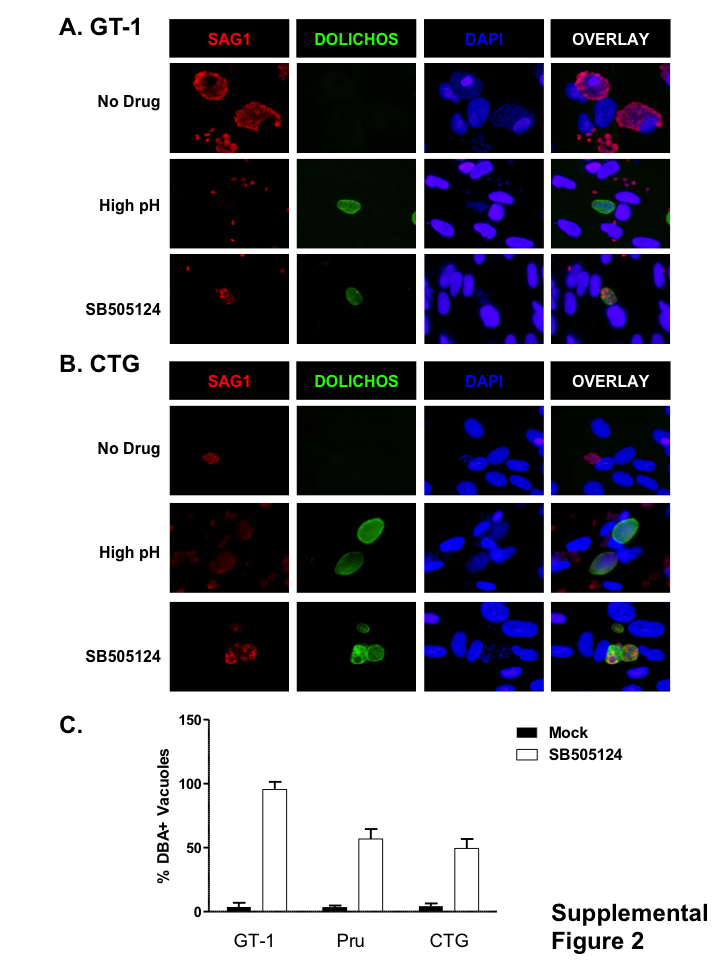

Supplement: Figure S2 — HFFs grown on glass coverslips were infected with GT-1 (A) or CTG (B) strain tachyzoites and then either mock-treated or treated with pH 8.2 media or 3 µM SB505124. After 72 h, cells were fixed and stained with anti-SAG1 and Dolichos-FITC to visualize cysts. Shown are representative images. C. Quantification of numbers of GT-1, Pru, and CTG strain parasites thath were Dolichos (DBA)+ after exposure to SB505124. Note that images for Pru strain parasites are shown in Figure 2B. (TIFF) [file ppat.1004180.s002.tiff]
