# Supplementary material for: Forward Genetic Screening Identifies a Small Molecule That Blocks Toxoplasma gondii Growth by Inhibiting Both Host- and Parasite-Encoded Kinases
Source: PLoS Pathog. 2014 Jun 12;10(6):e1004180. doi: 10.1371/journal.ppat.1004180 (PMC4055737; doi:10.1371/journal.ppat.1004180)
Supplement: Table S2 — List of either PCR primers to clone TgMAPK1 constructs or of RT-PCR primers to assess bradyzoite gene expression. (DOCX) [file ppat.1004180.s004.docx]

| **Table 2. Primers used in this study** | | | |
| --- | --- | --- | --- |
| 1. **Primers used for cloning TgMAPK1 Constructs** | | | |
| **Amplicon** | **Size (bp)** | **Forward Primer Sequence (5’-3’)** | **Reverse Primer Sequence (5’-3’)** |
| TgMAPK1^SBR^  Allelic Replacement | 944 | TGCATGGCGATGAGTTTCTGAACG | TCGTGTCGACGTTTCTTCTGTGGA |
| TgMAPK1^SBR^  Allelic Replacement Confirmation | 1055 | GGTCTCGAACTGTCCCTCAAAGTTCT | GCGGAATCAGTGAACGAACCCTGTA |
| TgMAPK1-HAx3 | 1557 | TACTTCCAATCCAATTTAATGCACTACTCGGCGTCGTACTCGGC | TCCTCCACTTCCAATTTTAGCCAGCTGCTGCTGGCCATGC |
| 1. **Primers used for Real-Time PCR** | | | |
| **Amplicon** | **Size (bp)** | **Forward Primer Sequence (5’-3’)** | **Reverse Primer Sequence (5’-3’)** |
| TgBAG1 | 214 | GGGATGTACCAAGCATCCTG | AGGGTAGTACGCCAGAGCAA |
| TgLDH2 | 183 | TGTAACAGGCATGCCAGAAG | TCGTTTCTGCTCCACTCCTT |
| TgENO1 | 336 | CGAGGGGTGGCTGAAAAAGTATCC | CAGCGAAGGCCCACGACAAG |
| TgENO2 | 356 | AGCGGCGCATCCACTGGCATCTAC | AACGGGACGGGCATTACCATCTTG |
| TgACT1 | 589 | ATGTATGTCGCTATCCAGGCCGTT | TGATCTTCATGGTGGAAGGAGCCA |
